# *Trichoderma* volatiles effecting *Arabidopsis*: from inhibition to protection against phytopathogenic fungi

**DOI:** 10.3389/fmicb.2015.00995

**Published:** 2015-09-29

**Authors:** Metwally Kottb, Tamara Gigolashvili, Dominik K. Großkinsky, Birgit Piechulla

**Affiliations:** ^1^Institute for Biological Sciences, University of RostockRostock, Germany; ^2^Biocenter, Botanical Institute and Cluster of Excellence on Plant Sciences, University of CologneCologne, Germany; ^3^Department of Plant and Environmental Sciences, Copenhagen Plant Science Centre, University of CopenhagenTaastrup, Denmark; ^4^Institute of Plant Sciences, University of GrazGraz, Austria

**Keywords:** *Trichoderma asperellum* IsmT5, *Botrytis cinerea*, *Alternaria brassicicola*, *Arabidopsis thaliana*, mVOCs, 6-pentyl-α-pyrone, glucosinolates, camalexin

## Abstract

*Trichoderma* species are present in many ecosystems and some strains have the ability to reduce the severity of plant diseases by activating various defense pathways via specific biologically active signaling molecules. Hence we investigated the effects of low molecular weight volatile compounds of *Trichoderma asperellum* IsmT5 on *Arabidopsis thaliana*. During co-cultivation of *T. asperellum* IsmT5 without physical contact to *A. thaliana* we observed smaller but vital and robust plants. The exposed plants exhibit increased trichome numbers, accumulation of defense-related compounds such as H_2_O_2_, anthocyanin, camalexin, and increased expression of defense-related genes. We conclude that *A. thaliana* perceives the *Trichoderma* volatiles as stress compounds and subsequently initiates multilayered adaptations including activation of signaling cascades to withstand this environmental influence. The prominent headspace volatile of *T. asperellum* IsmT5 was identified to be 6-pentyl-α-pyrone (6PP), which was solely applied to *A. thaliana* to verify the growth and defense reactions. Most noticeable is that *A. thaliana* preexposed to 6PP showed significantly reduced symptoms when challenged with *Botrytis cinerea* and *Alternaria brassicicola*, indicating that defense-activated plants subsequently became more resistant to pathogen attack. Together, these results support that products that are based on *Trichoderma* volatiles have the potential being a useful biocontrol agent in agriculture.

## Introduction

The genus *Trichoderma* (telemorph Hypocrea) includes cosmopolitan soil-borne species, some of them are saprophytes and are frequently isolated from soil and wood as well as plant litter (Błaszczyk et al., 2011). Other species were detected inside of root tissues of many plants as opportunistic, avirulent symbionts (Harman et al., 2004). *Trichoderma longibraciatum*, an example of a human pathogen, was isolated from infected tissues of immunocompromised individuals, who suffered opportunistic infections (Kuhls et al., 1999). Together, this reflects a large distribution and a pronounced adaptability of this genus to live in different habitats. Furthermore, *Trichoderma* species play an important role in the health of an ecosystem (Klein and Eveleigh, 1998) and since at least the 1930s species are known and used as biocontrol agents (plant growth promoting fungi) to reduce the severity of plant diseases (Weindling, 1932) and subsequently increase yields (Harman et al., 2004; Lorito et al., 2010). These beneficial effects were related to the control of deleterious soil microflora, the degradation of toxic compounds, the direct stimulation of root development by the production of phytohormones, enhanced solubility and subsequent increase in the availability of phosphorus and several micronutrients due to the presence of *Trichoderma* (Altomare et al., 1999; Gravel et al., 2007; Bae et al., 2009; Contreras-Cornejo et al., 2009; Martínez-Medina et al., 2011; Vos et al., 2015). Some effective *Trichoderma* strains were shown to produce a variety of microbe-associated molecular patterns (MAMPs) (Vinale et al., 2012). The first recognized MAMP was identified as an ethylene induced xylanase 2 (Xyn2/Eix) which is a potent elicitor of plant defense responses in specific tobacco and tomato cultivars (Rotblat et al., 2002). Plants colonized by *Trichoderma* species, or treated by e.g., cellulases, 18-mer peptaibols, harzianolide, and harzianopyridone, provide resistance to a wide variety of pathogenic microorganisms (Hermosa et al., 2012). According to Harman et al. (2004) each of the above mentioned activation process begins with the colonization of the plant roots by *Trichoderma* spp. *Trichoderma* species that are able to establish such interactions, induce massive changes in their transcriptome and metabolism (Reino et al., 2008; Brotman et al., 2010) and such metabolites have been found not only to directly inhibit the growth of pathogenic microorganisms but also increase disease resistance by triggering the defense system in plants (induced systemic resistance, ISR) (overview Vos et al., 2015). Furthermore, metabolite-pretreated plants responded to a pathogen attack much faster or more intensively (Verhagen et al., 2004; Shoresh et al., 2010; Verhage et al., 2010; Hermosa et al., 2012), a mechanism known as priming (Conrath, 2011).

The application of compounds originating from biological sources (biological control agents) and the development of novel sustainable crop protection strategies to reduce the usage of pesticides, bactericides and fungicides in agriculture are increasingly demanded by consumers. *Trichoderma* has the potential to find broad application, because it is already used due to its high mycoparasitic and antibiotic potential against different plant pathogens (review Vos et al., 2015). More than 60% of the registered biopesticides are based on *Trichoderma* (Verma et al., 2007).

We propose in a new and innovative hypothesis that volatile compounds emitted by *Trichoderma* facilitate the distribution of beneficial effects over long distances and that they may contribute to the improvement of plant growth. In the past decade progress has been made in understanding the role of microbial volatiles in multitrophic interactions and their potential functions (e.g., Kai et al., 2007, 2009; Vespermann et al., 2007; Minerdi et al., 2009; Wenke et al., 2010; Blom et al., 2011; Junker and Tholl, 2013; Naznin et al., 2013; D'Alessandro et al., 2014; Piechulla and Degenhardt, 2014). At present ca. 10,000 procaryots were identified, but only less than 500 bacterial and fungal species have been investigated regarding their potential to emit VOCs (Effmert et al., 2012; Lemfack et al., 2013). VOCs belong to different chemical classes, e.g., mono- and sesquiterpenes, alcohols, ketones, lactones, esters, thioalcohols, thioesters, and cyclohexanes (Splivallo et al., 2011; Kramer and Abraham, 2012; Lemfack et al., 2013). Due to their economical importance the volatile profiles of the prominent truffles were studied in detail and it was shown that the volatiles of *Tuber borchii, Tuber indicum*, and *Tuber melanosporum* inhibit leaf growth and root development of *A. thaliana* (Splivallo et al., 2007). Many fungi produce 1-octeno-3-ol, which enhances plant resistance to the necrotrophic fungus *Botrytis cinerea* by inducing defense signaling cascades (Kishimoto et al., 2007; Contreras-Cornejo et al., 2014). Volatiles of *Alternaria alternata, Penicillium charlesii*, and *Penicillium aurantiogriseum* promote growth and starch accumulation in several plant species (Ezquer et al., 2010).

As mentioned above, *Trichoderma* has numerous ways of indirectly enhancing plant growth (Vos et al., 2015) however so far very limited information is available upon volatile-based interactions in the context of plant growth promotion. *Trichoderma viride* for example stimulated the growth of *A. thaliana* in the absence of direct physical contact and increased lateral root formation and established early-flowering phenotypes (Hung et al., 2013). It was also shown that volatiles of *Trichoderma* act antibiotically against pathogenic fungi and thereby confer plant growth promotion (Vinale et al., 2008a). These hints stimulated us to screen several *Trichoderma* species for VOCs-mediated effects and that subsequently one specific strain was chosen to investigate thoroughly the morphological, physiological and molecular alterations in *A. thaliana* upon co-cultivation with *Trichoderma asperellum* IsmT5. These studies included the monitoring of growth and defense reactions in the plants under normal and challenged conditions.

## Material and methods

### Biological materials

The wild type *Arabidopsis thaliana* (Col-0) was used in all experiments. Seeds were kindly provided by Dr. Zhonglin Mou (Microbiology Department, University of Florida, Gainesville, FL, USA). Transgenic lines were obtained from: DR5::GUS from Dr. Zsuzsanna Kolbert (Department of Plant Biology, Faculty of Science and Informatics, University of Szeged, Szeged, Hungary), PDF1.2::GUS from Dr. Anja van Dijken (Utrecht University, The Netherlands), pYUC8::GUS from Dr. Stephan Pollmann (Metabolomics Unit at the Center of Plant Biotechnology and Genomics, Madrid, Spain), and PR1::GUS from the European Arabidopsis Stock Centre (UK; http://arabidopsis.info/).

*Trichoderma* species used in this study were isolated from different locations in Egypt during January 2005–January 2006. *T. asperellum* IsmT5 was isolated from the rhizosphere of maize cultivated in Ismailia, *Trichoderma harzianum* was isolated from okra roots cultivated in Serabium village, and *Trichoderma spp*. was isolated from soil in Sinai (Wadi El Arbeen). *T. asperellum* IsmT5 Samuels, Lieckf. and Nirenberg was identified and deposited at The Centraalbureau voor Schimmelcultures (Applied and Industrial Mycology/Identification Service CBS-KNAW Fungal Biodiversity Centre, Utrecht, The Netherlands) under accession number CBS 137093 (Data sheets 1 and 2 in Supplementary Material).

*Alternaria brassicicola* was obtained from Dr. Eckehard Koch (Julius-Kühn-Institute, Braunschweig, Germany) and *Botrytis cinerea* from Dr. Andreas v. Tiedemann (University of Göttingen, Germany).

### *Trichoderma*—plant co-cultivation

The effects of volatiles of *Trichoderma* sp. on plant growth were tested in a closed and open co-cultivation system (Figure S1A). *A. thaliana* seeds were surface sterilized (1 min 70% ethanol, 5 min 5% calcium hypochlorite, rinsed four times with sterilized distilled water) and cultivated on MS medium (Murashige and Skoog, 1962). The seeds were vernalized for 3 days at 4°C in the absence of light and then four seedlings were transferred to glass jars containing solidified MS medium in slant position for 3 days. At this time a disc (∅ 0.5 cm) of a 7 days old *Trichoderma* culture grown on nutrient broth agar (30 g glucose; 2 g NaNO_3_; 1 g KH_2_PO_4_; 1 g yeast extract; 2 g peptone; 0.5 g KCl; 0.5 g MgSO_4_·7H_2_O; 8 mg CaCl_2_·6H_2_O; 1 mg ZnSO_4_·7H_2_O; 10 mg FeSO_4_·7H_2_O per liter) (Bonnarme et al., 1997) at 20°C was introduced into a small beaker containing 20 ml broth agar. The small beaker was placed into the jar without any physical contact to the plants or MS agar. The jar was placed in 3 cm distance to the plants. The seedlings were exposed to *Trichoderma* volatiles for 9 days at 24°C and 84 μmolm^−2^s^−1^ of light at a 16/8 h light/dark cycle. Under these growth conditions *Trichoderma* did not produce spores and fungal growth was not observed in control experiments in MS agar. Fresh weight, leaf area and root length were determined four times (technical replicates). The experiments were repeated three times (biological replicates).

### Analysis of anthocyanins

The accumulation of anthocyanins in *Arabidopsis* seedlings was determined after 9 days of co-cultivation with *T. asperellum* IsmT5 applying the method of Neff and Chory (1998) with some modifications. At least two samples of 100 mg VOCs-exposed seedlings in comparison with control plants were incubated overnight in 150 μL of methanol acidified with 1% HCl (w/v). After the addition of 100 μL of distilled water, anthocyanins were separated from chlorophylls with 250 μL of chloroform. The absorbance of the aqueous phase was measured at 535 and 657 nm. The relative amount of anthocyanins per 100 mg of fresh weight was calculated by the equation *A*_535_–*A*_657_ × 100.

### Analysis of chlorophyll content

Total chlorophylls were extracted in 1 mL of 80% aqueous acetone containing 2.5 mM sodium phosphate buffer (pH 7.8) to minimize conversion of chlorophylls into phaeophytins. The suspension was centrifuged at 4°C at 2500 rpm for 10 min. For each experiment, at least two groups of 100 mg of seedlings (after 9 days of co-cultivation) were used and fluorescence was determined using a spectrometer (Ultrospec 3000, Pharmacia). The absorbance at different wavelength (480, 646, 647, 652, 663, 664, and 750 nm) was measured. The absolute amount of chlorophyll was calculated using the extinction coefficient indicated by Porra et al. (1989). Concentration of chlorophyll, expressed as μg/mg was determined by the following equations:

$$\begin{array}{l} \text{Chl a}=12.25\;(A_{663-750})\times 2.55\;(A_{647-750})\\ \text{Chl b}=20.31\;(A_{647-750})\times 4.91\;(A_{663-750})\\ \text{Chl a}+\text{b}=17.76\;(A_{647-750})+7.34\;(A_{663-750}) \end{array}$$

### Determination of trichome density

Trichome number was measured upon the appearance of the first true fully expanded rosette leaf produced by treated and untreated plants (after 9 days of co-cultivation). The target leaf was first removed from the plant and traced. The leaf area was measured using a leaf area meter (AM 300, ADC BioScientific, Hoddesdon Herts, UK) and the adaxial trichome number was determined under a dissecting microscope. The trichome density was calculated as trichome number per leaf area (number/cm^2^).

### Reactive oxygen species (ROS)

#### H_2_O_2_

Hydrogen peroxide content of treated and untreated *A. thaliana* seedlings (after 9 days of co-cultivation) was measured spectrophotometrically after reaction with potassium iodide (KI) according to Chakrabarty et al. (2009). The reaction mixture consisted of 0.5 ml supernatant of 0.1% trichloroacetic acid (TCA) seedling extract, 0.5 ml of 100 mM K-phosphate buffer (pH 7), and 2 ml reagent (1 M KI, w/v in fresh double-distilled water). 0.5 ml of 0.1% TCA served as control. After 1 h of incubation in darkness at room temperature, the absorbance was measured at 390 nm. The amount of hydrogen peroxide was calculated using a standard curve prepared with known concentrations of H_2_O_2_.

#### Viability of roots

Root activity is an indirect indicator of tissue viability, and can be determined by using 2, 3, 5-triphenyl tetrazolium chloride (TTC) (Shen et al., 1991). Viable (respiring) tissue reduces TTC to red-colored triphenyl formazan by accepting electrons from the mitochondrial electron transport chain (Comas et al., 2000). Thus, a decrease in root activity is an indication of reduced respiration and reduced viability often resulting from tissue damage. Briefly, 50 mg of freshly harvested root tissue (after 9 days of co-cultivation) was treated with 5 ml of 0.4% TTC solution (w/v) and 5 ml of 0.067 M phosphate buffer (pH 7.4). This mixture was incubated at 40°C for 3 h followed by the addition of 2 ml 2 M H_2_SO_4_. Thereafter, roots were ground in 10 ml ethyl acetate to extract red triphenyl formazan. Its concentration was measured spectrophotometrically at 485 nm and expressed as A_485_ g^−1^ h^−1^.

### Quantification of phytohormones and camalexin

Determination/quantification of the phytohormones ABA, indole-3-acetic acid (IAA), JA, SA, and the phytoalexin camalexin was performed after 9 days of co-cultivation according to Großkinsky et al. (2014) with slight modifications. Briefly, volatile exposed and control seedlings were frozen and ground in liquid nitrogen; 200 mg per sample were extracted with 80% methanol and internal standards were added for the quantification of camalexin (6-fluoroindole-3-carboxyaldehyde; Sigma-Aldrich, Steinheim, Germany) and the phytohormones (deuterium-labeled hormones; Olchemim Ltd, Olomouc, Czech Republic). The extracts were directly subjected to HPLC analysis for camalexin (Ultimate 3000; Dionex, Sunnyvale, USA). For phytohormone determination, methanol extracts were passed through Chromafix C18-columns (Macherey-Nagel, Düren, Germany), completely dried (Integrated SpeedVac® Concentrator System AES1000; Savant Instruments Inc., Holbrook, USA), resuspended in 20% methanol, passed through Chromafil PES-20/25 filters (Macherey-Nagel, Düren, Germany) and subjected to UHPLC-MS/MS (Thermo Scientific; Waltham, USA) analyses.

### Quantification of glucosinolates

Freeze-dried exposed (after 9 days of co-cultivation) and control seedlings (100 mg) were transferred into 2 mL reaction tubes and lyophylised. The isolation and analysis of GSL content was performed by using the desulpho-GSL method on an ultra-performance liquid chromatography (UPLC) device (Waters, Eschborn) as described in Frerigmann et al. (2012).

### GUS assay

Transgenic *Arabidopsis* lines carrying a GUS reporter system were used in this study. After 9 days of co-cultivation, the expression patterns of the ß-glucuronidase activity were elucidated by histochemical staining using at least 10 exposed and control seedlings, each (Jefferson et al., 1987). In order to visualize the GUS activity, whole seedlings were incubated in a solution of 1 mM X-gluc (5-bromo-4-chloro-3-indolyl-ß-D-glucuronic acid), 0.1 M phosphate buffer (pH 7.0), 10 mM EDTA, 0.1% (v/v) Triton X-100, and 1 mM K_3_Fe(CN)_6_ overnight at 37°C. Subsequently, seedlings were incubated in buffer and fixed for at least 20 min in a mixture of 5% (v/v) formaldehyde, 5% (v/v) acetic acid, and 20% ethanol (v/v), followed by an additional incubation in 50% (v/v) ethanol. The seedlings were preserved in formaldehyde containing 80% (v/v) ethanol till microscopic examination. Images of the plants were recorded with a digital camera Ricoh Cx4 (Ricoh, Tokyo, Japan).

### Head space collection and GC/MS analysis of *Trichoderma* VOCs

In a glass Petri dish of 9 cm diameter, a disc (∅ 0.5 cm) from a 7 days old *T. asperellum* IsmT5 culture was inoculated on 20 ml of broth agar (2.2). The Petri dish was placed into the incubation chamber of a slightly modified airflow collection system (Figure S1B) (Kai et al., 2007). Charcoal-purified, sterile humidified air was pushed through a closed system. Volatiles present in the headspace were carried along and finally trapped in a column containing 40 mg Super Q as trapping material (Alltech Associates, Deerfield, Illinois, USA). The airstream of the pump (Gardner Denver, Puchheim, Germany) was adjusted to a constant flow of 0.6 l min^−1^. The volatiles were collected in 24 h intervals during the incubation period of up to 9 days. Volatiles were eluted from the trapping material with 300 μl dichloromethane. For quantitation, 10 μl of nonyl acetate (5 ng) was added as an internal standard. Samples were analyzed using the Shimadzu GC/MSQP5000 (Kyoto, Japan) equipped with a DB5-MS column (60 m × 0.25 mm × 0.25 μm; J&W Scientific, Folsom, California, USA). Splitless injection of 1 μl sample was performed at 200°C with a sampling time of 2 min using a CTC autosampler (CTC Analytics, Zwingen, Switzerland). The initial column temperature was set at 35°C, followed by a ramp of 10°C min^−1^ up to 280°C with a final hold for 15 min at 280°C. Helium was used as the carrier gas at a flow rate of 1.1 ml min^−1^. Ionization was performed at 70 eV and mass spectra were obtained using the scan modus (2 scans per second, total ion count, 40–280 *m/z*). Confirmation of structure assignments was done by comparison of mass spectra and retention times with those of available standards, with literature data, as well as by comparison with spectra covered by the NIST107 (version 1998) library, and by comparison of Kovats indices. Experiments were replicated at least three times.

### Effect of 6PP on *Arabidopsis*

To investigate effects of 6PP (Sigma-Aldrich, Steinheim, Germany) on *Arabidopsis*, three concentrations (0.5, 1, and 2 mM) of the pure compound were applied. Briefly, 1 ml of 6PP was dissolved in sterilized distilled water and then applied to a small glass tube that was fixed in a 9 cm Petri dish distant to 3 days old seedlings. Petri dishes were sealed and incubated for 9 days at 24°C in vertical position. Fresh weight and root length were measured. The experiment was replicated four times.

The potential of 6PP in reducing disease severity of phytopathogenic fungi such as *A. brassicicola* and *B. cinerea* on *A. thaliana* was also tested. 2 mM 6PP (concentration equals the concentration emitted by *T. asperellum* IsmT5) were applied into the soil where 4 weeks old *A. thaliana* grew. After 24 h, four plant leaves in each glass jar were challenged with the pathogens. 5 μl of a spore suspension (5 × 10^6^ spores/ml) of *B. cinerea* or *A. brassiciola* were spotted onto the leaves. The percentage of disease severity (lesion size) was measured 5 days post infection.

### RT–PCR

Total RNA was extracted from leaves (100 mg FW) of exposed (after 9 days of co-cultivation) and control plants. After 9 days of volatile treatment with 2 mM 6PP (see Effect of 6PP on *Arabidopsis*) or co-cultivation with *T. asperellum* IsmT5 (see *Trichoderma*—Plant Co-cultivation) seedlings were harvested, cells were homogenized by grinding with mortar and pestle in liquid nitrogen and RNA was enriched using the Nucleospin kit (Machery-Nagel, Düren, Germany). mRNA was reverse-transcribed into cDNA using the primers (Table S1) and SuperScript reverse transcriptase (Thermo Scientific Maxima Reverse Transcriptase) according to the protocol of the manufacturer. Gene-specific primers (Table S1, obtained from Life Technologies, Carlsbad, USA) were used to amplify respective genes via PCR (Hippauf et al., 2010). The ubiquitin gene (AT4G05320) served as internal control.

### Statistics

F-statistics of ANOVA was used to calculate significance in all experiments. Each F-statistic is a ratio of mean squares. The numerator is the mean square for the term. The denominator is chosen such that the expected value of the numerator mean square differs from the expected value of the denominator mean square only by the effect of interest. The effect for a random term is represented by the variance component of the term. The effect for a fixed term is represented by the sum of squares of the model components associated with that term divided by its degrees of freedom. Therefore, a high F-statistic indicates a significant effect (Moreira et al., 2013).

## Results

### *Trichoderma* volatiles alter morphology and physiology of *Arabidopsis*

**Figure 1:**
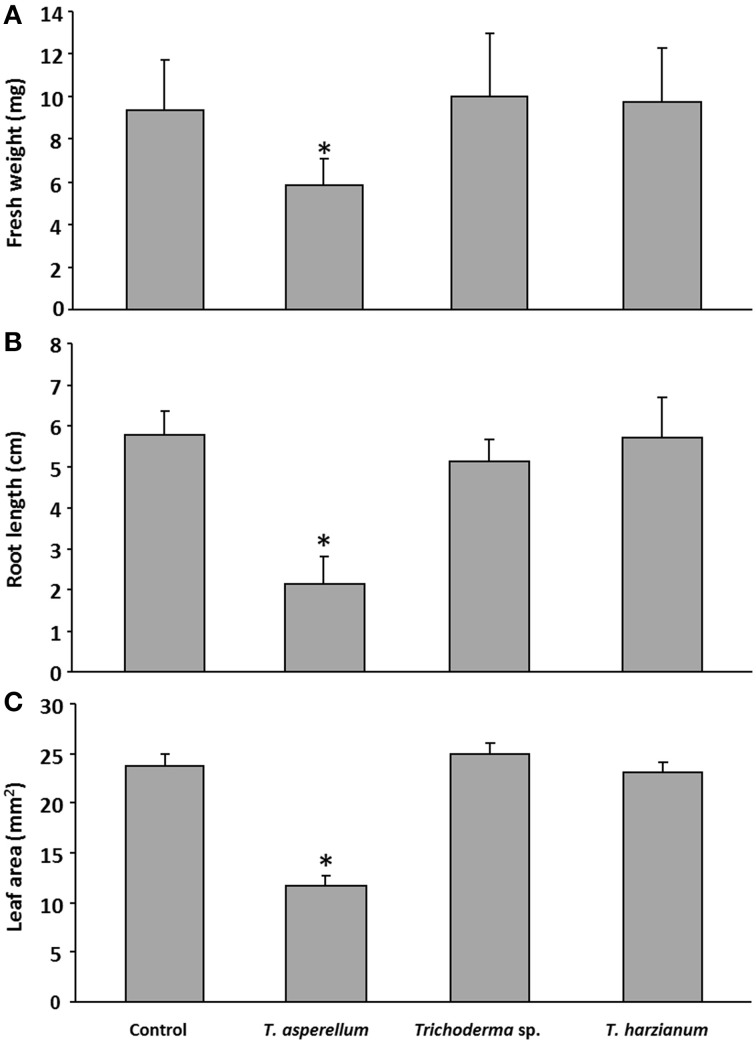
**Effect of volatiles of different *Trichoderma* isolates on the growth of *Arabidopsis thaliana***. Seedlings and fungi were co-cultivated for 9 days at 24°C and 84 μmol m^−2^s^−1^ of light at a 16/8 h light/dark cycle. Fresh weight **(A)**, root length **(B)**, and leaf area **(C)** were measured (four technical replicates). The experiments were repeated three times (biological replicates). Non-exposed control plants were also investigated, *n* = 12, error bars indicate SD, ^*^P < 0.05.

To investigate the impact of *Trichoderma* volatile compounds on plant growth, a simple and reliable co-cultivation system was established (Figure S1A). After 9 days of co-cultivation and concurrent exposure various parameters were investigated. First, we determined how growth and development of *A. thaliana* were influenced by three different *Trichoderma* isolates (Figure 1). The three isolates originated from different locations in Egypt. While *Trichoderma* sp. and *T. harzianum* volatiles did not influence the morphology and habitus of *A. thaliana*, exposure of seedlings to *T. asperellum* IsmT5 volatiles reduced fresh weight, root length, and leaf area by ca. 40, 60, and 50%, respectively (Figures 1A–C). Thus, *T. asperellum* IsmT5 volatiles were investigated in more detail.

**Figure 2:**
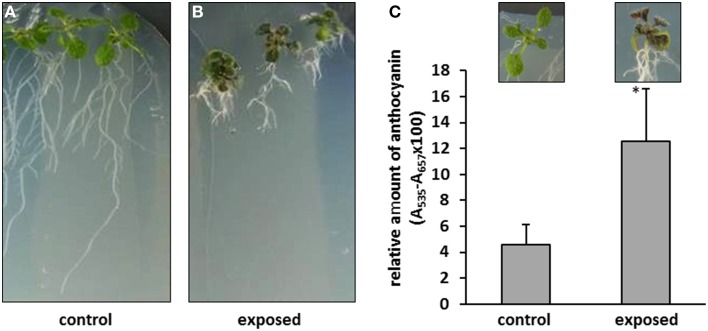
**Phenotype and anthocyanin accumulation in *Arabidopsis thaliana* exposed to *Trichoderma asperellum* volatiles**. Co-cultivation was performed for 9 days at 24°C and 84 μmol m^−2^s^−1^ of light at a 16/8 h light/dark cycle. Stunted but robust *Arabidopsis* seedlings were obtained after *Trichoderma* volatile exposure, control **(A)**, and exposed seedlings **(B)**. **(C)** Accumulation of anthocyanin in *Arabidopsis* seedlings co-cultivated with *Trichoderma asperellum* IsmT5: Anthocyanins were extracted from control (left) and exposed plants (right) and measured at 657 nm. Concentrations were calculated from three independent experiments and four technical replicates, *n* = 12, error bars indicate SD, ^*^P < 0.05.

**Figure 3:**
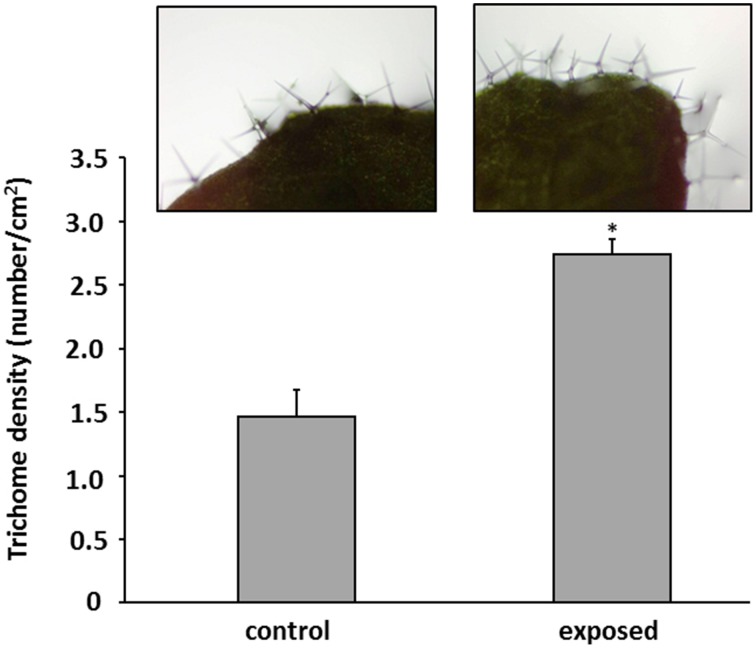
**Trichomes on leaves of *Arabidopsis thaliana* exposed to *Trichoderma asperellum* volatiles**. Co-cultivation was performed for 9 days at 24°C and 84 μmol m^−2^s^−1^ of light at a 16/8 h light/dark cycle. Trichomes were counted and leaf area was measured to determine trichome density (number per cm^2^) of control (left) and exposed plants (right). Density was calculated from five leaves, error bars indicate SD, ^*^P < 0.05.

The plants treated with *T. asperellum* IsmT5 volatiles appeared robust and showed no wilt or other detrimental symptoms (Figures 2A,B). Interestingly, the leaves of such exposed seedlings appeared to be much darker. Spectrophotometric analyses revealed a ca. three-fold higher accumulation of anthocyanin pigments in the co-cultivated seedlings compared to controls (Figure 2C). Since anthocyanin pigmentation plays a role in plant protection, e.g., forming a photoprotective screen in vegetative tissues and functioning as antimicrobial agent and feeding deterrent in the defense responses (Winkel-Shirley, 2001; Steyn et al., 2002) we further investigated morphological and physiological parameters of plant defense in *A. thaliana*. Microscopic examination of the leaf surface showed that trichome density was significantly increased by 47% in the leaves exposed to *T. asperellum* IsmT5 volatiles (Figure 3).

**Figure 4:**
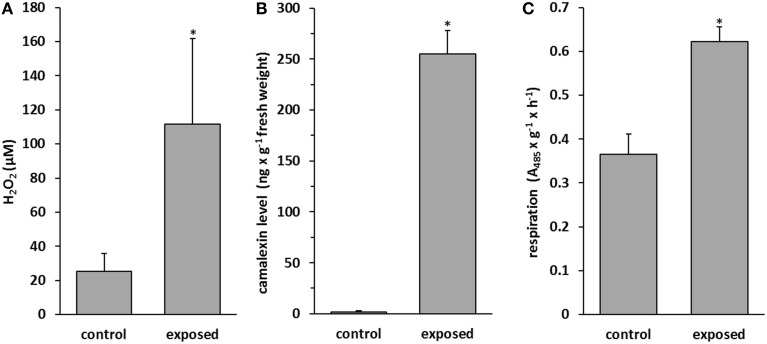
**Determination of defense molecules in *Arabidopsis thaliana* exposed to *Trichoderma asperellum* volatiles**. Co-cultivation was performed for 9 days at 24°C and 84 μmol m^−2^s^−1^ of light at a 16/8 h light/dark cycle. H_2_O_2_ **(A)**, camalexin **(B)**, and activity of respiration chain in roots **(C)** were measured in control and volatile exposed seedlings. Parameters were obtained from two independent experiments and five leaves, error bars indicate SD, ^*^P < 0.05.

Reactive oxygen species (ROS) are small molecules such as hydrogen peroxide (H_2_O_2_), superoxide anion (O${}_{2}^{•-}$), and hydroxyl radical (^•^OH) and are one of the earliest signals that activate plant defense responses (Singh et al., 2010). We examined H_2_O_2_ accumulation after 9 days of co-cultivation of *T. asperellum* IsmT5 with *A. thaliana*. The level of H_2_O_2_ was increased four-fold in the exposed plants compared to control plants (Figure 4A). Camalexin is a phytoalexin, which usually accumulates after pathogen attack or after treatment with abiotic elicitors such as UV or silver nitrate (Glawischnig, 2007). Camalexin accumulation increased by 97% in *A. thaliana* upon fungal volatile exposure (Figure 4B, Figure S2). Furthermore, tissue viability of volatile exposed seedlings of *A. thaliana* was investigated by determining mitochondrial respiration activity. Figure 4C documents a ca. 40% higher respiration activity in plants treated with *T. asperellum* IsmT5 volatiles referring to an increased viability in exposed seedlings. In summary, *A. thaliana* plants exposed to *Trichoderma* volatiles manifest improved survival strategies and defense responses.

### *Trichoderma* volatiles modulate phytohormone levels in *Arabidopsis*

**Figure 5:**
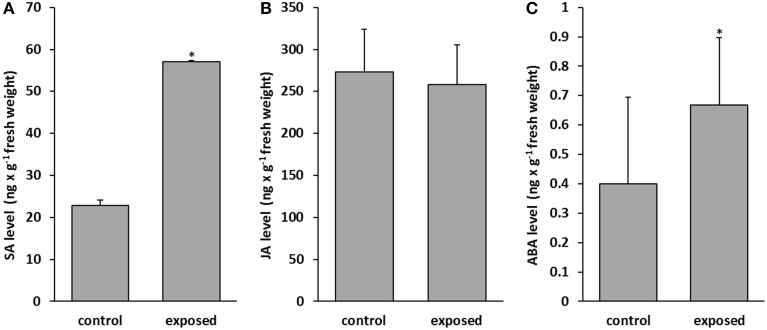
**Determination of phytohormones in *Arabidopsis thaliana* exposed to *Trichoderma asperellum* volatiles**. Co-cultivation was performed for 9 days at 24°C and 84 μmol m^−2^s^−1^ of light at a 16/8 h light/dark cycle. Salicylic acid **(A)**, jasmonic acid **(B)**, and abscisic acid **(C)** were measured in control (left column) and volatile exposed (right column) seedlings. Parameters were obtained from two independent experiments and five leaves, error bars indicate SD, ^*^P < 0.05.

Phytohormones are well known to regulate growth and development of plants and several plant hormones also act as central players in triggering the plant immune signaling network (Howe and Jander, 2008; Bari and Jones, 2009; Katagiri and Tsuda, 2010; Vos et al., 2015). Very little is known how the presence of volatiles is translated or mediated via phytohormone depending signaling cascades. Here we addressed the main signaling pathways. The levels of three prominent phytohormones were examined in control and *T. asperellum* IsmT5 volatile-exposed seedlings. The results showed that in co-cultured plants salicylic acid (SA) was increased by 61% and abscisic acid (ABA) by 40% (Figures 5A,C). No significant differences were recorded for other hormones, e.g., jasmonic acid (JA) (Figure 5B). Apparently the signal transduction chains that stimulate SA and ABA accumulation were selectively activated by the fungal volatiles leading to increased phytohormone levels.

### *Trichoderma* volatiles stimulate gene expression in *Arabidopsis thaliana*

**Figure 6:**
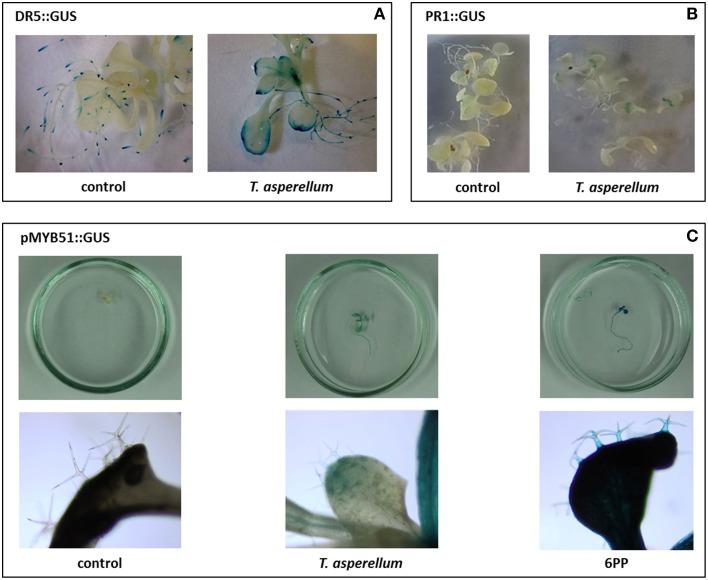
**Expression of genes in leaves of transgenic *Arabidopsis thaliana* lines exposed to *Trichoderma asperellum* volatiles**. Transgenic cell lines equipped with promoter gus constructs were co-cultivated with *Trichoderma asperellum* IsmT5. Promoters originated from the DR5 (indole biosynthesis) **(A)** and PR1 (pathogenesis related protein 1) **(B)**, and MYB51 (transcription factor involved in trichome production) **(C)**. Co-cultivation was performed for 9 days at 24°C and 84 μmol m^−2^s^−1^ of light at a 16/8 h light/dark cycle. Glucuronidase assay was performed in control and volatile exposed seedlings. Blue color indicates the expression of the *uid* gene in the tissue.

In order to investigate whether *T. asperellum* IsmT5 volatiles cause gene activation in *Arabidopsis*, we used four transgenic lines with promoter-*uidA* chimeric gene constructs related: (i) to IAA responses (DR5::GUS and YUC8::GUS), (ii) to the transcriptional activation of indolic glucosinolates (MYB51::GUS), (iii) plant defensin (PDF1.2::GUS), and (iv) pathogenesis related protein 1 (PR1::GUS). In exposed seedlings all four promoters were activated and a distinct blue coloration compared to control plants was observed (Figure 6, Figure S3). The promoter of the DR5 gene was primarily activated at the rim of the leaves (Figure 6A), while the PR1 promoter revealed gus activation in a zone near the base of the leaf (Figure 6B). Interestingly, the MYB51 promoter was activated in the trichomes and epidermal tissue (Figure 6C). PDF 1.2::GUS and YUC8::GUS were expressed in whole leaves of the exposed seedlings (Figures S3A,B). In summary, *Arabidopsis* plants respond to *T. asperellum* IsmT5 volatiles by activating genes that are involved in phytohormone biosynthesis and plant defense.

### Analysis of volatile profiles of *Trichoderma* species

The results described above demonstrate that volatiles released by *T. asperellum* IsmT5 caused changes at the morphological, physiological, and transcriptional level of *A. thaliana*. Since the volatile profile of *T. asperellum* IsmT5 was so far unknown, we collected and analyzed headspace volatiles (Figure S1B). In the GC chromatogram three peaks could be identified (Figure S4A). The contribution of the peak area of peak number #4 reflects ca. 90% of the total volatile emission. By comparison of mass spectra, retention times, and Kovats indices, the compound of peak #1 was tentatively identified as 1-octen-3-ol, #2 as nonanal and #4 as 6PP (Table 1). Because of its abundance, we focused our future work on compound #4. Its chemical identity was verified by comparison with the commercially available 6PP (Figures S4C,D). The dynamics of volatile emission was recorded in 24 h intervals throughout of 10 days (Figure S5). A maximum of 6PP accumulation (450 ng/μl = 2.7 mM) was reached at day 8 of cultivation.

**Table 1:** **Volatile organic compounds in the headspace of *Trichoderma asperellum* IsmT5**.

**Number in Figure 8A**	**Volatile compound**	**Retention time (min)**	**Kovats index**	**% Similarity to database**
#1	1-octen-3-ol	12.733	967	87
#2	nonanal	15.142	1090	87
#3	n-nonyl acetate (internal standard)	18.517	1287	89
#4	6-pentyl-2-pyrone	21.042	1453	not available in NIST 107, version 1998

### Effects of 6-pentyl-α-pyrone on *Arabidopsis thaliana*

**Figure 7:**
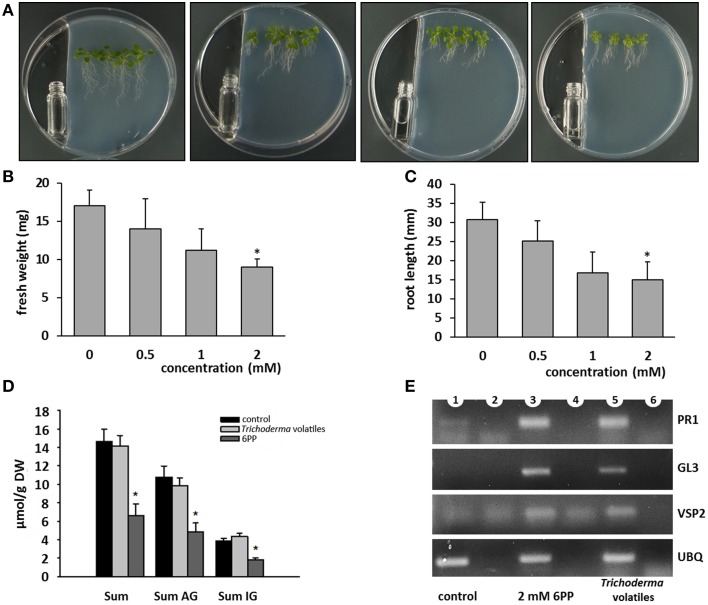
**Effects of 6-pentyl-α-pyrone (6PP) on the growth, glucosinolate level, and defense gene expression of *Arabidopsis thaliana***. One ml of 6PP of different concentrations were filled into sterile glass vials and positioned next to 3 days old *A. thaliana* seedlings growing on MS medium **(A)**. After 9 days fresh weight **(B)** and root length **(C)** were recorded. The experiment was repeated for three times and four seedlings were placed on the agar, *n* = 3, error bars indicate SD, ^*^P < 0.05. **(D)**: 3 days old *A. thaliana* seedlings were co-cultivated with *T. asperellum* IsmT5 (see *Trichoderma*—Plant Co-cultivation) or exposed to 6PP (see Effect of 6PP on *Arabidopsis*). After 9 days of co-cultivation or after 9 days of 6PP application whole seedlings were harvested and glucosinolates were extracted and analyzed by HPLC (see Quantification of Glucosinolates). AG, aliphatic glucosinolates; IG, indolic glucosinolates. *n* = 3, error bars indicate SD, ^*^P < 0.05. **(E)**: Via RT-PCR the expression of defense genes were analyzed: PR1, pathogenesis related protein 1; GL3, transcription factor GLABRA 3; VSP2, vegetative storage protein. Expression of ubiquitin (UBQ) was used for standardization. Controls in lane 2, 4, 6: RT-PCR without polymerase.

After identification of the major volatile compound released by *T. asperellum* IsmT5, we determined which concentration of 6PP caused morphological alterations in *A. thaliana*. Two milliliters of 0.5 mM, 1 mM, and 2 mM were placed in a vial next to *Arabidospsis* seedlings (Figure 7A). Exposure to 2 mM 6PP revealed ca. 50% reduction of fresh weight and root length (Figures 7B,C, respectively). Surprisingly, four aliphatic and three indole glucosinolates were also reduced upon 6PP and *Trichoderma* volatile application (Figure 7D; Figures S6A,B). To our knowledge it was shown for the first time that a volatile compound influenced glucosinolate levels in plants.

We furthermore investigated the effects of *T. asperellum* IsmT5 volatiles and 6PP on the expression (RT-PCR) of defense related genes in exposed *Arabidopsis* seedlings. Gene expression of the SA-induced pathogen related protein PR-1, the transcription factor involved in trichome formation GL3, and VSP2 activated by ethylene was clearly induced upon both treatments indicating that various defense genes were up-regulated upon volatile exposure (Figure 7E).

**Figure 8:**
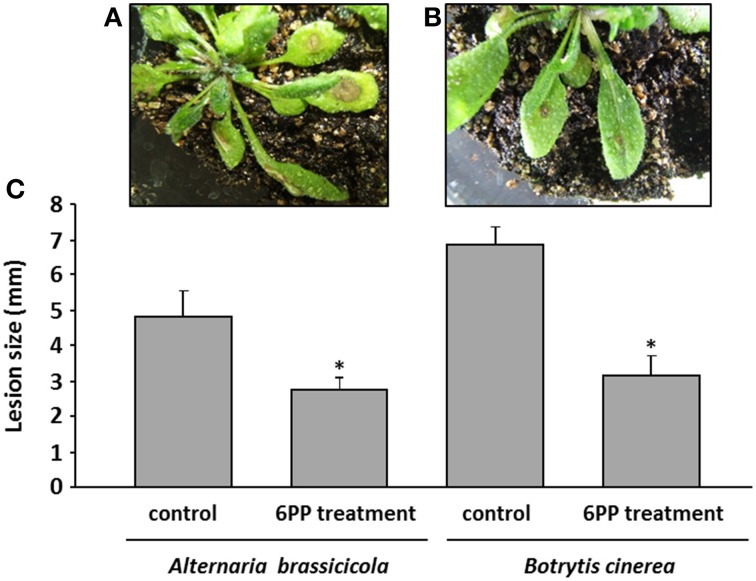
**Development of disease symptoms in *Arabidopsis thaliana* leaves after preincubation with 6-pentyl-α-pyrone (6PP)**. Four weeks old *Arabidopsis* plants were grown in glass containers (see Effect of 6PP on *Arabidopsis*). Twenty milliliters of 2 mM 6PP were applied to the soil of each glass jar. The jar was covered with a lid and sealed with Parafilm®. Twenty-four hours later 50 μl of *Botrytis cinerea* spore suspension or of *Alternaria brassicicola* (each 5 × 10^6^ spores/ml) were applied to the leaves. Representative examples of *A. thaliana* plants treated with a spore suspension of *B. cinerea* are shown in the upper panel **(A)**: without preexposure to 6PP = control; **(B)**: with 6PP preexposure. Lesions are visible as brown spots on the leaves. At the 5th day after inoculation the diameter of the lesions were recorded and calculated **(C)**. The experiment was repeated for three times, each with four plants, *n* = 3, error bars indicate SD, ^*^P < 0.05.

To further investigate whether the volatile compound 6PP is a plant defense inducer, we performed the following experiment: *Arabidopsis* plants were pretreated with 2 mM 6PP for 24 h followed by either the application of a spore suspension of the phytopathogenic fungus *A. brassicicola* or *B. cinerea*. After 5 days of incubation the lesion sizes were recorded. In both treatments the symptom sizes were reduced by ca. 40% and ca. 60%, respectively (Figure 8). We concluded that the exposure to 6PP induced resistance against fungal pathogens in *Arabidopsis*. A direct effect of 6PP on *A. brassicicola* was also investigated and spore germination was significantly reduced by 2 mM 6PP (Figure S7).

## Discussion

The goal was to study morphological and physiological reactions of *A. thaliana* upon exposure to volatiles of four *Trichoderma* species. Only *T. asperellum* IsmT5 showed significant effects on plant growth and was therefore selected for further investigations. The fungal volatiles did not kill the plant but initiated several plant defense responses like the production of ROS, increased trichome number, accumulation of anthocyanins, and the phytoalexin camalexin. Furthermore, the expression of e.g., SA pathway depending defense genes were increased. We analyzed the headspace volatiles of *T. asperellum* IsmT5 and identified the most prominent VOC in the spectrum to be 6PP, which has a strong coconut-like aroma. Here we demonstrated a dual function of 6PP, (i) preincubation of *A. thaliana* with 6PP improved its resistance to the phytopathogenic fungi *Botrytis* and *Alternaria* (indirect action) and (ii) reduction of spore germination of *Alternaria* (direct action). These results support that *Trichoderma* volatiles have the potential being a useful biocontrol agent in agriculture.

### Test system

Several co-cultivation systems were developed to test effects of fungal volatiles on plant growth. Naznin et al. (2013) and Hung et al. (2013) used airtight cultivation systems and observed growth promotion of tobacco and *A. thaliana* by *Phoma* and *T. viride*, respectively. We prepared slanted agar medium in glass jars for plant growth and inserted a beaker with fungi to avoid direct contact of both organisms (Figure S1A). This method had several advantages, e.g., easy and rapid inspection of the phenotypic responses of the roots and shoots during exposure to microbial volatiles, and no limitation to use only seedlings. We tested two alternative ways to cover the jars, either laying the lid on the jar however without sealing with Parafilm (closed system), or a funnel with sterile glass wool at the tip was placed upside down (open system) (Figure S1A). We preferred to use the open system (i) because it reflects the natural situation more closely, and (ii) to overcome the problem of CO_2_ accumulation due to metabolic activity of the microorganism, since it was recently shown that CO_2_ accumulated to 10-fold higher levels compared to ambient levels in closed containers (Kai and Piechulla, 2009).

Three different *Trichoderma* strains/isolates were investigated regarding their effects on *Arabidopsis* seedlings. Interestingly, the strains induced different phenotypic alterations in *A. thaliana*. The volatiles of *T. asperellum* IsmT5 influenced the plants negatively, which was manifested in the inhibition of primary root, reduction in size of leaves and fresh weight. In contrast, plant growth promotions due to *Trichoderma* volatiles have been observed by Hung et al. (2013) and Contreras-Cornejo et al. (2014). These contradictory results are most likely due to the different (open vs. closed) test systems used.

### Headspace analysis and 6-pentyl-α-pyrone application

The defense responses initiated in *A. thaliana* upon *Trichoderma* volatile exposure prompted us to investigate the nature of the bioactive compound. Identification of headspace volatiles indicated different chemical classes i.e., alcohols, alkanes, and pyrones (Table 1). Similar compound diversity (25 different compounds) was also previously reported for *Trichoderma atroviride* (Stoppacher et al., 2010). Very prominent levels of 6PP were detected in the headspace of *T. asperellum* IsmT5. This compound and other α-pyrone analogs have been detected in cultures of *T. viride* (Collins and Halim, 1972), *T. harzianum* (Claydon et al., 1987), *T. koningii* (Simon et al., 1988), *T. atroviride* (Reithner et al., 2005), *T. citrinoviride* and *T. hamatum*, (Jeleñ et al., 2013), and recently in *T. asperellum* 328 (Wickel et al., 2013).

Lactones are generally very pleasant and potent flavor compounds (Kapfer et al., 1989) and a variety of microorganisms perform *de novo* lactone biosynthesis (Tressl et al., 1978). 6PP has a coconut-like aroma and it was known for long time that this odor appeared during *Trichoderma* cultivation (Bisby, 1939; Rifai, 1969). The emission of 6PP by *T. asperellum* IsmT5 reached levels of up to 450 ng/μl (= 2.7 mM), similar concentrations were reported by Kalyani et al. (2000) and Serrano-Carreón et al. (2004). A systematic optimization of growth conditions improved the production of 6PP by three orders of magnitude in *T. atroviride* (Oda et al., 2009). This was a success because of its use as a perfume in food and cosmetic industries. Beside the technological application it is well known that many natural lactones have antibacterial, antifungal or anti-inflammatory biological activity (Claydon et al., 1987; Simon et al., 1988; Cooney and Lauren, 1999; Pezet et al., 1999; Romero-Guido et al., 2011).

### Plant defense reactions

The interaction of *Trichoderma* with a plant was thought to start by colonization of the outer root layers, resulting in the induction of resistance mechanisms to prevent further colonization (Yedidia et al., 1999; Harman et al., 2004; Mukherjee et al., 2013; Vos et al., 2015). Here we demonstrate that a volatile based interaction between *A. thaliana* and *T. asperellum* IsmT5 also exists, which might occur prior to physical contact. Although many evidences exist that *Trichoderma* activates plant immunity and development through different mechanisms, it was so far unknown whether microbial VOCs play a role in any of these plant defense processes (Contreras-Cornejo et al., 2014).

Here it was shown for the first time that *Arabidopsis* exposed to *T. asperellum* IsmT5 volatiles doubled its trichome number (Figure 3). Trichome formation in plants is a general defense strategy primarily developed to hinder landing, moving, and penetration of insects and other organisms on plant surfaces. The presented results indicate that the volatiles are apparently perceived as an intervening organismal interaction. While detailed investigations are needed to understand the underlying mechanisms related to these morphological alterations *T. asperellum* IsmT5 volatiles also induced typical plant defense responses at the physiological level, such as increasing the H_2_O_2_ level in leaves (Figure 4A). Similar observations were recorded by Splivallo et al. (2007). They found that truffle volatiles induced an oxidative burst in *Arabidopsis*. Increase of ROS in *A. thaliana* was also found upon bacterial volatile exposure (Wenke et al., 2012). Contradictory results of H_2_O_2_ production induced by *Trichoderma* volatiles were reported by Hung et al. (2013) and Contreras-Cornejo et al. (2014). Another early response in pathogen defense is the oxidation by peroxidases, subsequently reducing the amount of oxygen diffusing from the roots into the surrounding environment (Tiwari et al., 2002). This mechanism helps plant roots to avoid the uptake of toxic materials and thereby provides protection (Singh et al., 2007). The roots of volatile exposed plants showed increase in such root activity (Figure 4C). Similar results were recorded in rice and of seedlings of *Ageratina adenophora* (Zhang et al., 2012). Since ROS trigger many downstream processes leading to a dynamic defense responses characterized by inhibition of the growth of invaders through phytoalexin formation, callose deposition, strengthening of cell walls, synthesis of secondary metabolites and pathogenesis related (PR) proteins (Xu et al., 2008; Vinale et al., 2008b; Shoresh et al., 2010), we hypothesize that *T. asperellum* IsmT5 volatiles are perceived as oxidative stress and thus inducing alterations in the antioxidant enzyme machinery and accumulation of other protective substances in *A. thaliana*. A striking observation was the dark coloration of *Trichoderma* volatiles exposed leaves (Figure 2C) resulting from anthocyanin accumulation. Such accelerated anthocyanin accumulation due to volatile stress has to our knowledge not yet been reported although these results fit very well to the known function of anthocyanin to act as antimicrobial agents and feeding deterrents (Winkel-Shirley, 2001; Steyn et al., 2002; Shin et al., 2013). Furthermore, camalexin known as an important phytoalexin of *Arabidopsis* and an integral part of the plant defense system in *A. thaliana* (Glawischnig, 2007) was significantly up-regulated during volatiles exposure (Figure 4B). As other phytoalexins, camalexin production can be elicited by bacterial and fungal phytopathogens (as well as abiotic stress) and possesses antimicrobial activity (Großkinsky et al., 2012). Contreras-Cornejo et al. (2011) proved that *Arabidopsis* seedlings colonized with *T. virens* or *T. atroviride* accumulated high levels of camalexin. Camalexin deficient mutants such as *pad3* (encodes last step in phytoalexin biosynthesis) displayed enhanced susceptibility to *B. cinerea* (Ferrari et al., 2003), and in *A. thaliana* treated with *T. atroviride pad3* was up-regulated in roots and leaves (Salas-Marina et al., 2011). In this study the accumulation of camalexin due to *T. asperellum* IsmT5 volatile exposure highlights a new aspect and showed that not only by direct contact between plant and *Trichoderma* camalexin is induced but also by air borne signals. Glucosinolates play central roles in plant/biotic interactions and they are important determinants for plant fitness in the field. Hydrolysis products of glucosinolates are active against a wide variety of organisms, such as insects, plants, fungi, and bacteria (Vaughn, 1999). In this study we analyzed the levels of glucosinolates in *Arabidopsis* plants co-cultivated with *T. asperellum* IsmT5 or exposed to the fungal volatile 6PP. Our results demonstrate that 6PP caused a significant reduction in the accumulation of glucosinolates, while levels were not significantly altered in plants co-cultivated with *T. asperellum* IsmT5 (Figure 7D). Whereas the catabolism of glucosinolates and *de novo* biosynthesis is well balanced in plants co-cultivated with *T. asperellum* IsmT5, the degradation seems to be more dominant in 6PP-treated plants. Apparently, other volatiles of *T. asperellum* IsmT5 spectrum prevent a reduction of the essential glucosinolates and consequently without weakening the plant defense system. The function of Trp-derived indole glucosinolates in *Arabidopsis* immunity was validated with infection phenotypes of *cyp81F2* and *penetration2* (*pen2*) mutants. Analyses of loss-of-function mutant of *pen2* (an alternative myrosinase) suggested that PEN2-mediated glucosinolate metabolism is important for pre-invasive resistance, while camalexin is contributing at the post-invasive stage of immunity (Lipka et al., 2005; Hiruma et al., 2010; Sanchez-Vallet et al., 2010; Schlaeppi et al., 2010). These observations are also in agreement with the activation of the SA pathway of *T. asperellum* Ism5 co-cultivated plants as shown in (Figure 5) and MYB51 has been previously reported to be important regulator of glucosinolates at SA-signaling (Frerigmann and Gigolashvili, 2014). We therefore expected the increase in the levels of pathogen related genes such as DR5::GUS, YUC8::GUS, PDF1.2::GUS, PR1::GUS, MYB51::GUS (Figure 6 and Figure S3). These results further supported that fungal volatiles were perceived as stress, which initiated defense processes and improved plant immunity.

The defense network in plants is regulated by the action of plant hormones via two main mechanisms, systemic acquired resistance (involvement of SA) and induced systemic resistance (regulated by ethylene and JA). It became clear that an intensive interplay between hormone signaling pathways exists, which effectively determines the response to specific types of invader. The activation of phytohormone signaling cascades by direct interactions between *Trichoderma* spp. and *A. thaliana* or other plants was already demonstrated to trigger JA or SA dependent systemic resistance (Contreras-Cornejo et al., 2011; Salas-Marina et al., 2011; Velázquez-Robledo et al., 2011; Yoshioka et al., 2012; Vos et al., 2015), however volatile based activations add a new facette to such strategies. While SA and ABA accumulated in *A. thaliana* exposed to *T. asperellum* IsmT5 volatiles, levels of JA were not altered (Figure 5). Why, in contrast, the marker gene for the JA/ethylene mediated signaling in *A. thaliana*, PDF 1.2a, was expressed in transgenic lines (Figure S3) could be explained by a species-specific activation of the signaling cascades as found in *T. hamatum* T382 and *T. asperelloides* (Mathys et al., 2012; Brotman et al., 2013).

The presented results show that *A. thaliana* perceives the *Trichoderma* volatiles as stress compounds and subsequently initiates multilayered (morphological, physiological, and gene expression level) adaptations and activations of signaling cascades to withstand this environmental influence. This hypothesis was supported when the major volatile compound of the VOC spectrum of *T. asperellum* Ism5 was identified and *A. thaliana* preincubated with the pure compound 6PP and challenged with the phytopathogenic fungi *Botrytis* and *Alternaria*. The leaves showed significantly less necrotic symptoms compared to untreated plants (Figure 8). Consequently it was concluded that the volatiles of *T. asperellum* IsmT5 or 6PP activate the accumulation of typical defense molecules such as ROS, camalexin, anthocyanins, and the SA dependent plant hormone pathways and subsequently defense-activated plants become more resistant to pathogen attack and exhibited smaller lesions. Vinale et al. (2008a) also reported a reduction of disease symptoms in pea, tomato, and canola seedlings after addition of purified secondary metabolites. This effect correlated with elevated expression of chitinase, PR1 protein, and endochitinase. Maize plants growing in soil that was drenched with 6PP for 4 days prior to inoculation with *Fusarium moniliforme* showed considerable suppression of seedling blight compared to untreated controls (El-Hasan and Buchenauer, 2009). Cutler et al. (1986) were the first who recorded inhibitory effects on wheat coleoptiles. In addition, 6PP acts directly on spore germination of *A. brassicicola* (Figure S7), also demonstrated by Intana and Chamswarng (2007) and Yenjit et al. (2008) for *A. brassicicola* and *Phythium aphanidermatum*, respectively. Taken together, volatile metabolites from *T. asperelleum* IsmT5 (6PP) are involved in direct and indirect interactions between fungi and plants thereby have the potential to influence biocontrol processes (Cottier and Mühlschlegel, 2012). Deciphering the signaling cascades in plants that are induced by *Trichoderma* volatiles is a future challenge. So far, to the best of our knowledge, only one example is known which showed that a WRKY transcription factor is part of the signaling cascade in bacterial volatile plant interactions (Wenke et al., 2012). In the future, further investigations at the molecular level are required to shed light on the role of *Trichoderma* volatiles, especially 6PP, and bacterial volatiles in activation of defense mechanisms in plants to improve plant resistance and to design plant protection systems.

### Conflict of interest statement

The authors declare that the research was conducted in the absence of any commercial or financial relationships that could be construed as a potential conflict of interest.

## Abbreviations

VOCs: Volatile organic compounds
6PP: 6-pentyl-α-pyrone
gus: glucoronidase
ROS: reactive oxygen species
SA: salicylic acid
JA: jasmonic acid
ABA: abscisic acid.
