# Supplementary material for: Trichoderma volatiles effecting Arabidopsis: from inhibition to protection against phytopathogenic fungi
Source: Front Microbiol. 2015 Sep 29;6:995. doi: 10.3389/fmicb.2015.00995 (PMC4586454; doi:10.3389/fmicb.2015.00995)
Supplement: Supplementary file 1 [file DataSheet1.PDF]

Institut für Biowissenschaften
Attn.: Metwally Kottb
Albert-Einstein-Str 3
Biochemie
Univerität Rostock
18059 Rostock
Germany

Utrecht, November 21, 2013

CBS IDENTIFICATION SERVICE

Your reference: **e-mail d.d. 17-02-2013**

Our reference: **Det 13.140** Please state always our reference number when you contact us.

Herewith we inform you about the result of our identification of your strain(s).

Trichoderma asperellum Samuels, Lieckf. & Nirenberg

Isolate is grown on MEA for 4 days at 25°C and genomic DNA was extracted using the Ultraclean microbial DNA isolation kit (MoBio, Solana Beach, CA) according to the manufacturer's instructions. Amplification of the ITS regions, including ITS-1, ITS-2 and the 5.8S rDNA gene (primers V9G and LS266) and the Elongation factor (primers EF1-728f and TEF1-LLErev) are done as described in the following publications:
-de Hoog & Gerrits van den Ende: Molecular diagnostics of clinical strains of filamentous Basidiomycetes (Mycoses 41, 1998).
-Mascleux F.: Phylogenetic relationships of human-pathogenic Cladosporium (Xylohypha) species inferred from partial LS rRNA sequences (Journal of Medical and Veterinary Mycology 33, 1995)
- Carbone & Kohn: A method for designing primer sets for speciation studies in filamentous ascomycetes. (Mycologia 91, 1999)
- Jacklitsch et al: Hypocrea voglmayrii sp. nov. from the Austrian Alps represents a new phylogenetic clade in Hypocrea/Trichoderma (Mycologia 97, 2005)
Sequencing reactions were performed with the Big Dye Terminator Cycle Sequencing Ready Reaction kit and carried out for both strands. Sequencing reactions were purified by gel filtration through Sephadex G-50 (Amersham Pharmacia Biotech, Piscataway, NH), equilibrated in double-distilled water and analyzed on the ABI PRISM 310 Genetic Analyzer (Applied Biosystems).

The invoice for this identification will be sent separately.

With kind regards,

T. van Doorn, Bsc.
