# Supplementary material for: Trichoderma volatiles effecting Arabidopsis: from inhibition to protection against phytopathogenic fungi
Source: Front Microbiol. 2015 Sep 29;6:995. doi: 10.3389/fmicb.2015.00995 (PMC4586454; doi:10.3389/fmicb.2015.00995)
Supplement: Supplementary file 2 [file DataSheet2.PDF]

CBS FUNGAL BIODIVERSITY CENTRE
An institute of the Royal Netherlands Academy of Arts and Sciences

Executing a PCR with ribosomal DNA

1. Subject

To execute a restriction analysis or sequencing, some parts of the ribosomal DNA is used. By means of so called primers a selected part of the ribosomal DNA is amplified. This reaction is called PCR (polymerase chain reaction).

2. Principle

The ribosomal DNA contains repeats, that consist of a small subunit (SSU, 18S), an internal transcribed spacer (ITS1), 5.8S, an internal transcribed spacer (ITS2) and the large subunit (LSU, 26S). These repeats are separated by NTS, non-transcribed spacers.

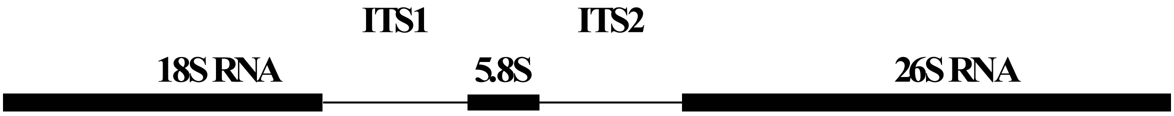

With two primers, mostly a 17- to 20-mer sequence, part of the ribosomal DNA is selected and elongated by means of an enzyme, DNA polymerase. For this purpose the DNA should be single stranded. DNA will become single stranded at high temperature (denaturation). The principal of PCR is the repetitive cycle of denaturation, polymerisation and renaturation.

3. Reagents and material

3.1 electrophoresis-unit
3.2 bromophenolbleu (Biorad 161-0404)
3.3 sucrose (Merck 7653)
3.4 primer NS1 (5′-gTAgTCATATgCTTgTCTC-3′)
3.5 primer NS7 (5′-gAggCAATAACAggTCTgTgATgC-3′)
3.6 primer NS24 (5′-AAACCTTgTTACgACTTTTA-3′)
3.7 primer ITS1 (5′-TCCgTAggTgAACCTgCgg-3′)
3.8 primer ITS4 (5′-TCCTCCgCTTATTgATATgC-3′)
3.9 primer ITS5 (5′-ggAAgTAAAAgTCgTAACAAgg-3′)
3.10 primer 5.8SR (5′-TCgATgAAgAACgCAgC-3′)
3.11 primer LR7 (5′-TACTACCACCAAgATCT-3′)
3.12 primer V9 (5′-TgCgTTgATTACgTCCCTgC-3′)
3.13 primer NS7 (5′-gAggCAATAACAggTCTgTgATgC-3′)
3.14 primer V9G (5′-TTACgTCCCTgCCCTTTgTA-3′)
3.15 primer 5.8G (5′-AATgTgCgTTCAAAgATTCg-3′)
3.16 primer LS266 (5′-gCATTCCCAAACAACTCgACTC-3′)
3.17 primer Oli 4 (5′-CTggTTgATYCTgCCAgT-3′)
3.18 primer Oli 16 (5′-CYgCAggTTCACCTACRg-3′)
3.19 ultrapure dNTP set 100 mM (Pharmacia 27-2035-01): 2′-deoxyadenosine-5′-triphosphate, 2′-deoxythymidine-5′-triphosphate, 2′-deoxycytidine-5′-triphosphate en 2′-deoxyguanosine-5′-triphosphate
3.20 DNA-polymerase 5U/µl (SphaeroQ, TP05c; Gentaur, 1121040)
3.21 potassium chloride, KCl (Merck 4936)
3.22 Tris(hydroxymethyl)-aminomethane (Merck 8382)
3.23 magnesiumchloride hexahydrate, $MgCl_2$-6H_2O (Merck 5833)
3.24 gelatine (Merck 4078)
3.25 Triton X-100 (Merck 8603)

A.H.G. Gerrits van den Ende

ISO certificate scope: Accession, preservation, storage and supply of micro-organisms (public deposits, save deposits and patent deposits) and related information KEMA CERTIFICAAT ISO 9001:2000 RAAD VOOR ACCREDITATIE C 013
Postal address: P.O.Box 85167, 3508 AD Utrecht, The Netherlands. Telephone + 31 (0)30 2122600. E-mail: info@cbs.knaw.nl
Visiting address: Uppsalalaan 8, 3584 CT Utrecht, The Netherlands. Telefax + 31 (0)30 2512097 CBS homepage www.cbs.knaw.nl

3.26 mineral oil (Sigma M 3526)
3.27 chloric acid HCl, 1N (Ferak 11448)
3.28 sodium hydroxide, NaOH (Merck 6498)
3.29 glycerol 87%, (Merck 4093)
3.30 Na-EDTA (Titriplex III, Bio Rad 161-0729)
3.31 dithiotreitol, DTT (Sigma D-0632)
3.32 eppendorfcups 1.5 ml (Sarstedt 72.690)
3.33 Biozym Low marker 50, 100, 150, 200, 250, 300, 350, 400, 450, 500, 600, 700, 800, 900 en 1000 bp (Biozym 110000)
3.34 Promega 1 Kb marker 250, 500, 750, 1000, 1500, 2000, 2500, 3000, 4000, 5000, 6000, 8000 en 10.000 bp (Promega G5711)
3.35 Eurogentec SmartLadder (Eurogentec MW-1700-02) 200, 400, 600, 800, 1000, 1500, 2000, 2500, 3000, 4000, 5000, 6000, 8000, 10000 bp resp. 20, 40, 60, 80, 100, 15, 20, 25, 30, 40, 50, 60, 80, 100 ng/band
3.36 PCR vials ultra thin 200 µl (Biozym 179401)

4. Solutions

4.1 PCR-buffer 10x:
Add 9.32 g potassium chloride [3.21], 3.03 g Tris [3.22] and 1.53 g magnesiumchloride hexahydrate [3.23] to 220 ml ultrapure water. Adjust pH to 8.3 by adding ± 10 ml 1N HCl [3.27]. Add 0.25 g gelatin [3.24] and 2.5 ml Triton X-100 [3.25], heat the solution for 30 min. at 56°C. Adjust the total volume to 250 ml with ultrapure water. Make aliquots of 50 ml.

4.2 0.05M Tris pH 8.3:
Add 6.05 g Tris [3.22] to 500 ml ultrapure water. Adjust pH to 8.3 by adding ± 20 ml 1N HCl [3.27]. Add ultrapure water to a volume of 1000 ml.

4.3 1M Tris:
Dissolve 121.1 g Tris [3.22] in a total volume of 1000 ml ultrapure water.

4.4 1M Tris pH 8.0:
Dissolve 121.1 g Tris [3.22] op in 800 ml ultrapure water. Adjust pH to 8.0 by adding 1N HCl [3.27]. Add ultrapure water to a total volume of 1000 ml.

4.5 0.5M EDTA pH 8.0:
Add 186.1 g EDTA [3.30] to 800 ml ultrapure water and stir on a magnetic stirrer. Add while stirring ± 20 g sodium hydroxyde [3.28]. The pH will be approx. 8.0. Adjust total volume to 1000 ml with ultrapure water.

4.6 1M DTT:
Dissolve 1.54 g DTT [3.31] in 10 ml ultrapure water. Aliquot in portions of 1 ml and store at -20°C.

4.7 dNTP-mix 5 mM:
Pipet 50 µl dATP, 50 µl dTTP, 50 µl dCTP, 50 µl dGTP [3.19] and 800 µl ultrapure sterile water into a sterile eppendorfcup (3.32). Dilute this solution to 1 mM before use (50 µl mix [4.7] and 200 µl ultrapure sterile water).

4.8 DNA-polymerase dilutionbuffer:
Mix 0.2 ml 1M Tris, pH 8.0 [4.4], 0.2 ml 0.5M EDTA pH 8.0 [4.5], 0.1 ml 1M DTT [4.6], 49.5 ml ultrapure water and 50 ml glycerol 87% [3.29] thoroughly. Store solution at 4°C.

4.9 DNA-polymerase user solution 1 unit/µl:
Mix 20 µl DNA-polymerase 5 units/µl [3.20] with 80 µl DNA-polymerase dilutionbuffer [4.8] and vortex. Store solution at -20°C.

4.10 Loadingbuffer:
Dissolve 100 mg bromophenolbleu [3.2] in a few drops of ethanol. Add 10 ml ultrapure water. Dissolve 10 g sucrose [3.3] separately in 40 ml ultrapure water. Mix both solutions and store at 4°C.

4.11 TE-buffer:
Add 0.12 g Tris [3.22] and 0.04 g Na-EDTA [3.30] to 80 ml ultrapure water. Adjust pH at 8.0 with 1N HCl [3.27]. Heat the solution if EDTA doesn't dissolve very well. Adjust volume to 100 ml. Aliquot the solution in 50 ml tubes. Autoclave the solution for 15 min. at 121°C. Store at roomtemperature.

A.H.G. Gerrits van den Ende

Postal address: P.O.Box 85167, 3508 AD Utrecht, The Netherlands. Telephone + 31 (0)30 2122600. E-mail: info@cbs.knaw.nl
Visiting address: Uppsalalaan 8, 3584 CT Utrecht, The Netherlands. Telefax + 31 (0)30 2512097 CBS homepage www.cbs.knaw.nl

4.12 Low Range marker-solution:
Pipet 20 µl low range marker [3.33], 20 µl loading buffer [4.10] and 60 µl TE-buffer pH 8.0 [4.11] and mix. Use 6-8 µl marker-solution per lane.

4.13 1Kb marker-solution:
Pipet 30 µl Promega 1Kb-marker [3.34], 3 µl loading dye 6x and 27 µl TE-buffer pH 8.0 [4.11] and mix. Use 3-5 µl marker-solution per lane.

4.14 SmartLadder:
The SmartLadder of Eurogentec (3.35) is user ready. Per lane 5 µl is used. With this marker the size and the concentration of the amplicon can be estimated.

5. Protocol

5.1 Total PCR-mastermix for N samples [6.5]: (N+1)*30 µl ultrapure water, (N+1)*5 µl PCR-buffer [4.1], (N+1)*10 µl dNTP-mix 1x [4.7], (N+1) µl primer 1, (N+1) µl primer 2 and (N+1) µl DNA-polymerase user solution [4.9] and vortex [8.1].

5.2 Pipet 2 µl DNA of each sample in a seperate cup [3.36].

5.3 Add 48 µl of the mastermix [5.1] to each sample, creating a total volume of 50 µl.

5.4 Pipet 2-3 drops of mineral oil [3.26] in each cup, if PCR machine has no heated lid.

5.5 Choose a program for PCR according to 8.2.

5.6 Check the PCR on agarose gel. Pipet 2 µl loading buffer [4.10] on parafilm and add 8 µl PCR product (without oil).

5.7 Pipet the total volume of 10 µl into a slot.

5.8 Choose an appropriate marker and pipet on 2-3 different positions on gel.

5.9 Run electrophoresis at 75V, until DNA/bromophenolbleu has migrated into the gel. Continue with 150V for 1.5-2 hours. The voltage remains constant [6.3].

5.10 Check the gel at UV light, make a picture of the gel and/or write down the fragment sizes. This can be done with the ImageMaster VDS system (protocol 18, p. 61).

5.11 If PCR has succeeded, preparations can be made to start ARDRA or sequencing [6.4].

6. Remarks

6.1 The PCR-amplicons [5.9] can be electrophoresed at 150 V immediately. A nicer result is created by running at 50 V during 10 min. and increase voltage to 125 V.

6.2 A suitable marker [5.8] for fragment sizes between 50 and 1000 bp is Biozym Low Range marker [4.12]; for fragment sizes between 250 and 10.000 Promega 1 Kb marker [4.13] can be used.

6.3 The start and end values of the electrophoresis process depends on the size of the gel trays.

6.4 To obtain an amplicon of the ITS region including 5.8S, primers V9G en LS266 are used in a concentration of 50 pmol/µl. To get a SSU amplicon primers Oli 4 and Oli 16 are used with a double concentrations (100 pmol/µl), because of the modifications present in these primers.

6.5 In a 1.5 ml reaction vial a PCR mastermix to a maximum of 30 samples can be mixed.

7. Literature

7.1 http://www.protocol-online.net/Protocol.htm

7.2 Sambrook et al.

A.H.G. Gerrits van den Ende

8. Graphs and Tables

8.1 Pipetting protocol to make a mastermix for PCR

	1 sample	N samples
PCR buffer 10x	5 µl	(N+1)*5 µl
Ultrapure sterile H_2O	30 µl	(N+1)*30 µl
dNTP 1 mM	10 µl	(N+1)*10 µl
Primer 1 (...) 50 pmol/µl	1 µl	(N+1)*1 µl
Primer 2 (...) 50 pmol/µl	1 µl	(N+1)*1 µl
DNA polymerase 1 U/µl[1]	1 µl	(N+1)*1 µl

[1] the amount of units depends on the brand

8.2 Creating a PCR program depending on the used primer combinations

PCR cycle	Temperature in °C	Time in min:sec
Initial denaturation	94-98	2:00-10:00
First cycle step: denaturation	94-98	0:15-1:00
Second cycle step: primer annealing	V9G-LS266 50-56<br>ITS1-ITS4 48-52<br>NS1-NS24 48-52<br>Oli4-Oli16 48-52	0:15-1:00
Third cycle step: elongation	68-72	1:00-3:00
Final elongation	68-72	2:00-10:00

A frequently used PCR program at CBS:

Initial denaturation:	94°C	5 min.
35 cycli	94°C	45 sec.
	52°C	30 sec.
	72°C	2 min.
Final elongation:	72°C	6 min.

A.H.G. Gerrits van den Ende

Postal address: P.O.Box 85167, 3508 AD Utrecht, The Netherlands. Telephone + 31 (0)30 2122600. E-mail: info@cbs.knaw.nl
Visiting address: Uppsalalaan 8, 3584 CT Utrecht, The Netherlands. Telefax + 31 (0)30 2512097 CBS homepage www.cbs.knaw.nl
