# Supplementary material for: Trichoderma volatiles effecting Arabidopsis: from inhibition to protection against phytopathogenic fungi
Source: Front Microbiol. 2015 Sep 29;6:995. doi: 10.3389/fmicb.2015.00995 (PMC4586454; doi:10.3389/fmicb.2015.00995)
Supplement: Supplementary file 3 [file DataSheet3.DOCX]

**Supplement**

**Fig. S1: (A)** **Co-cultivation of *Arabidopsis thaliana* and *Trichoderma* spp. without direct contact.**

Four *Arabidopsis* plants were grown in glass containers (Weck®) on 40 ml half-strength MS agar for 9 days*. Trichoderma* was cultivated in 20 ml of broth medium in a small beaker. To avoid physical contact the beaker was placed in ca. 3 cm distance to the MS agar. Left: Lid was placed on the jar corresponding to a covered or closed system. Right: A funnel was placed on the jar. The tip was closed with sterile cotton. This system corresponds to an open system.

**(B)** **Volatile collection system**. Charcoal purified and humidified air was pumped over the *Trichoderma* culture growing on 20 ml broth in a Petri dish. Volatiles of the headspace were adsorbed on Super Q, followed by an elution with dichloromethane and analysis by GC-MS. **1**: activated charcoal filter, **2**: sterile cotton filter, **3**: 0.85% saline, **4**: glass container harboring a 9 cm Petri dish, **5**: glass column contain 40 mg adsorbant (Super Q), **6**: suction pump adjusted to 4.5 ml/min flow rate. Iso-Versinic (Roth GmbH, Germany) tubing was used to minimize system immanent volatile emission.

**Fig. S2 Determination of camalexin in *Arabidopsis thaliana* seedlings exposed to *Trichoderma asperellum***

Seedlings and fungi were co-cultivated for 9 days at 24°C and 84 μmol m^−2^s^−1^ of light at a 16 h/8 h light/dark cycle. HPLC chromatogram of extract of non-exposed *A. thaliana.* (**A**) Camalexin was extracted with methanol from *A. thaliana* seedlings and analysed via HPLC (**B**). The camalexin peak eluted at Rt = 11 min, excitation wavelength: 318 nm, emission wavelength: 370 nm.

**Fig. S3: Expression of genes in leaves of transgenic *Arabidopsis thaliana* lines exposed to *Trichoderma asperellum* volatiles**

*A. thaliana* transgenic cell lines equipped with promoter gus constructs were co-cultivated with *Trichoderma*. Promoters originated from PDFa1,2 (plant defensin) (A) and YUC8 (transcription factor involved in indole and JA biosynthesis) (**B**) genes. Co-cultivation was performed for 9 days at 24°C and 84 μmol m^−2^s^−1^ of light at a 16 h/8 h light/dark cycle. Glucuronidase assay was performed in control and volatile exposed seedlings. Blue colour indicates the expression of the *uid* gene in the tissue.

**Fig. S4: Verification of 6PP in the headspace of *Trichoderma asperellum***

Typical GC chromatogram (**A**) of *Trichoderma asperellum* IsmT5. #1 1-octen-3-ol, #2 nonanal, #3 nonayl-acetate (internal standard) and #4 6-pentyl-α-pyrone (6PP). The mass spectrum of compound 4 of (A) is shown in (**B**). Typical GC chromatogram of the commercially obtained 6PP (**C**). The mass spectrum of the commercially obtained 6PP of (C) is shown in (**D**).

**Fig. S5: Headspace volatiles of *Trichoderma asperellum* and emission of 6-pentyl-α-pyrone during fungal growth**

*T. asperellum* IsmT5 was grown in the VOC collection system (Fig. S1B).

The accumulation of 6PP in headspace by *T. asperellum* was recorded for 10 days. The columns present the amounts of 6PP accumulated within a 24 h interval.

**Fig. S6 Determination of glucosinolates in *Arabidopsis thaliana* exposed to *Trichoderma asperellum* volatiles or with** **6-pentyl-α-pyrone (6PP)**

*A. thaliana* seedlings were co-cultivated with *T. asperellum* IsmT5 (see 2.2) or exposed to 6PP (see 2.11). After 9 days of co-cultivation or 9 days of 6PP application whole seedlings were harvested and glucosinolates were extracted and analysed by HPLC (see 2.8), aliphatic glucosinolates (AG) (**A**) and indolic glucosinolates (IG) (**B**): 3-methylsulfinylpropyl-glucosinolate (3MSOP), 4-methylsulfinylbutyl-glucosinolate (4MSOB), 5-methylsulfinylpentyl-glucosinolate (5MSOP), 8-methylsulfinyloctyl-glucosinolate (8MSOO), indol-3-ylmethyl-glucosinolate (I3M), 4-methoxyindol-3-ylmethyl-glucosinolate (4MOI3M) were determined. n=3, error bars indicate SD, * significance P <0.05.

**Fig S7: Effects of 6-pentyl-α-pyrone (6PP) on spore germination of *Alternaria brassicicola***

1 ml of 6PP (pure solution and different concentrations) together with 10 ml broth medium (liquid) in a test tube where inoculated with 10 μl (= 10^6^ spores) *Alternaria* spore suspension. After  24 h  the number of germinated spores were counted (100% = 20 germinated spores). The

experiment was repeated  3 times, error bars indicate SD, * significance P <0.05.

Table S1. Primers used for RT-PCR analyses and *Trichoderma* identification

| gene | primer sequence 5’ to 3’ direction | stimulation by |
| --- | --- | --- |
| PDF1.2 FW | TCACCCTTATCTTCGCTGCTC | JA |
| PDF1.2 RV | TGTAACAACAACGGGAAAATAAACA | JA |
| PR1 FW | CTCGGAGCTACGCAGAACAACT | SA |
| PR1 RV | TTCTCGCTAACCCACATGTTCA | SA |
| VSP2 FW | ATGCCAAAGGACTTGCCCTA | ET |
| VSP2 RV | CGGGTCGGTCTTCTCTGTTC | ET |
| YUC 8 FW | CGTCTCAAGCTTCACCTTCC | IAA |
| YUC 8 RV | AGCCACTGGTCTCATCGAAC | IAA |
| GL3 FW | ATGGCTACCGGACAAAACAG |  |
| GL3 RV | CCTTCACCAATGTTGAAGACG |  |
| Ubiquitin FW | TGGTCAGTAATCAGCCAGTTTGG |  |
| Ubiquitin RW | GCACCACAAATACTTGACGAACAG |  |
| V9G | TTACGTCCCTGCCCTTTGTA |  |
| LS266 | GCATTCCCAAACAACTCGACTC |  |
| EF1 | ATGGGTAAGGA(A/G)GACAAGAC |  |
| EF2 | GGA(G/A)GTACCAGT(G/C)ATCATGTT |  |
