# Supplementary figures and images for: Trichoderma volatiles effecting Arabidopsis: from inhibition to protection against phytopathogenic fungi
Source: Front Microbiol. 2015 Sep 29;6:995. doi: 10.3389/fmicb.2015.00995 (PMC4586454; doi:10.3389/fmicb.2015.00995)

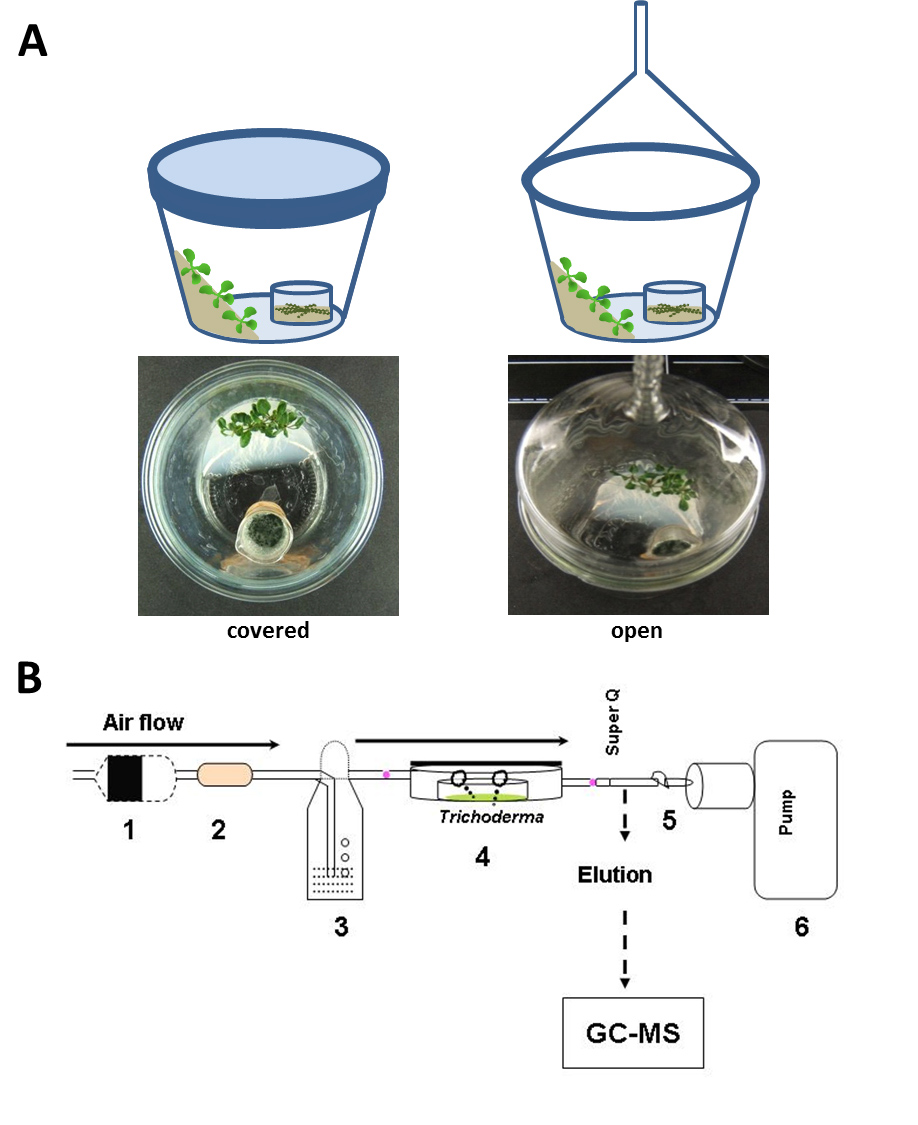

Supplement: Supplementary file 4 [file DataSheet4.ZIP › Data4/S1.JPEG]

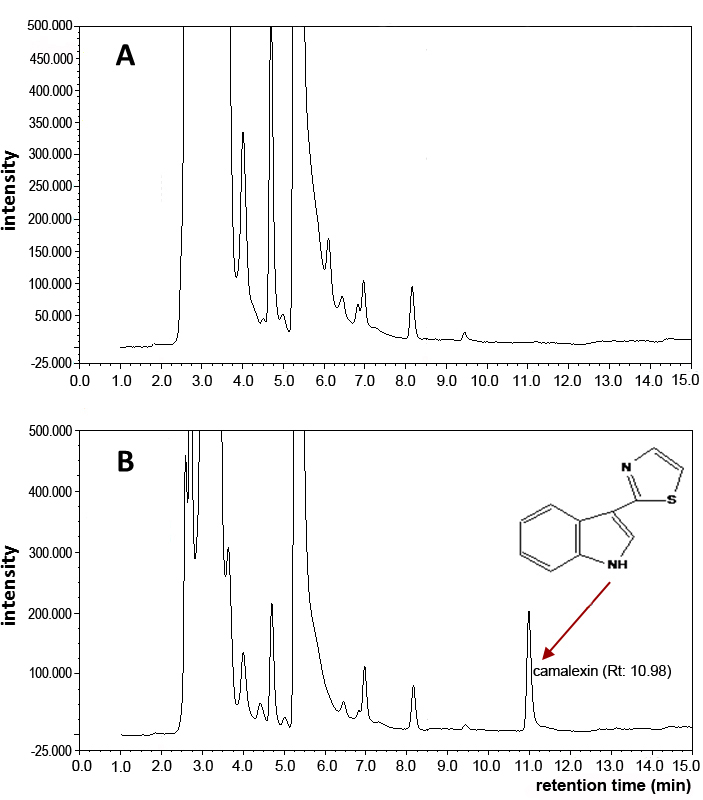

Supplement: Supplementary file 4 [file DataSheet4.ZIP › Data4/S2.JPEG]

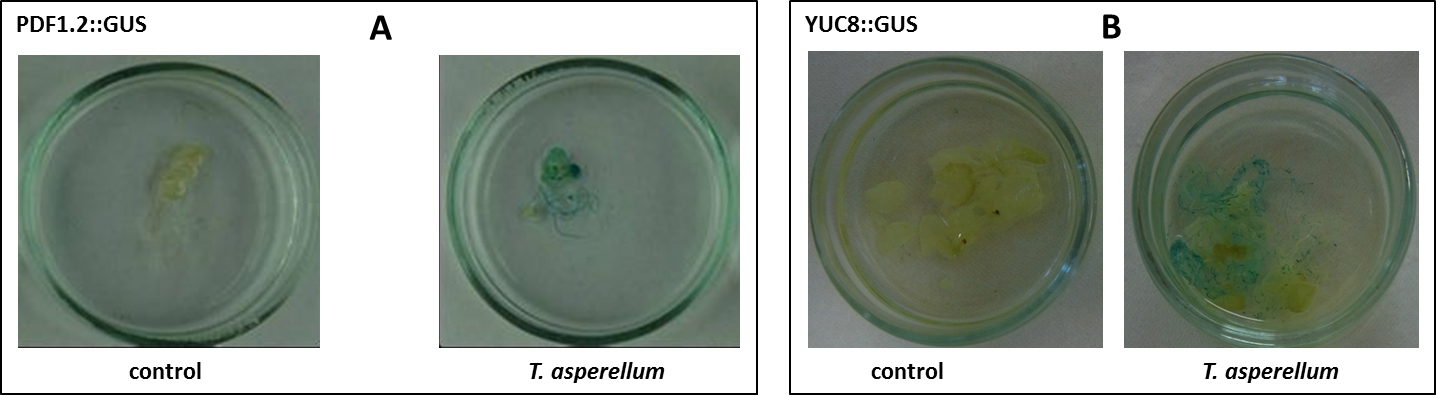

Supplement: Supplementary file 4 [file DataSheet4.ZIP › Data4/S3.JPEG]

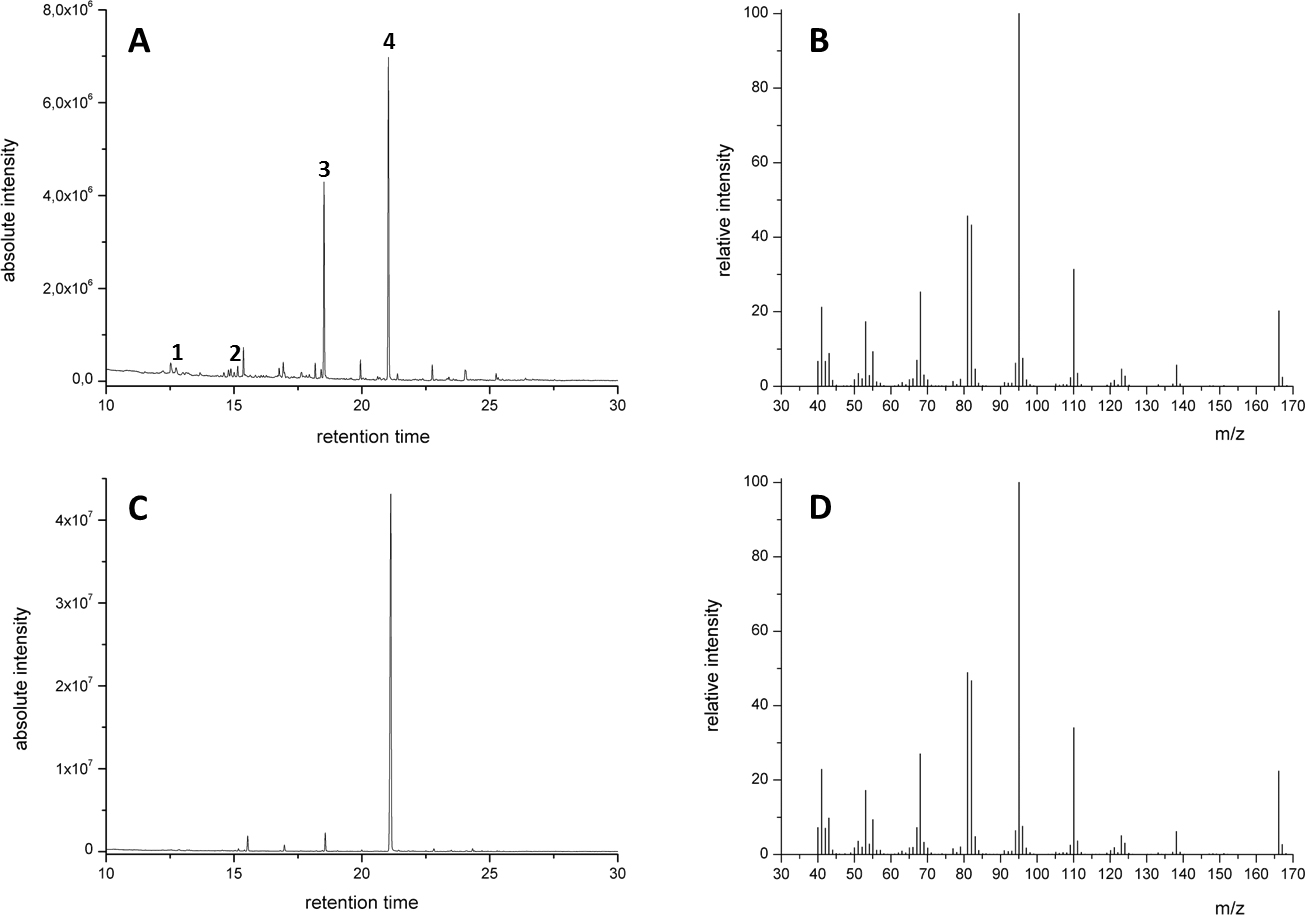

Supplement: Supplementary file 4 [file DataSheet4.ZIP › Data4/S4.JPEG]

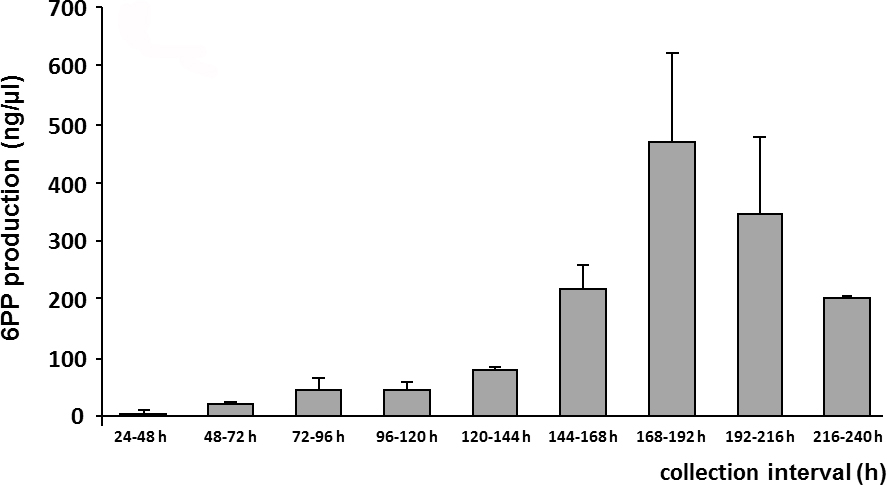

Supplement: Supplementary file 4 [file DataSheet4.ZIP › Data4/S5.JPEG]

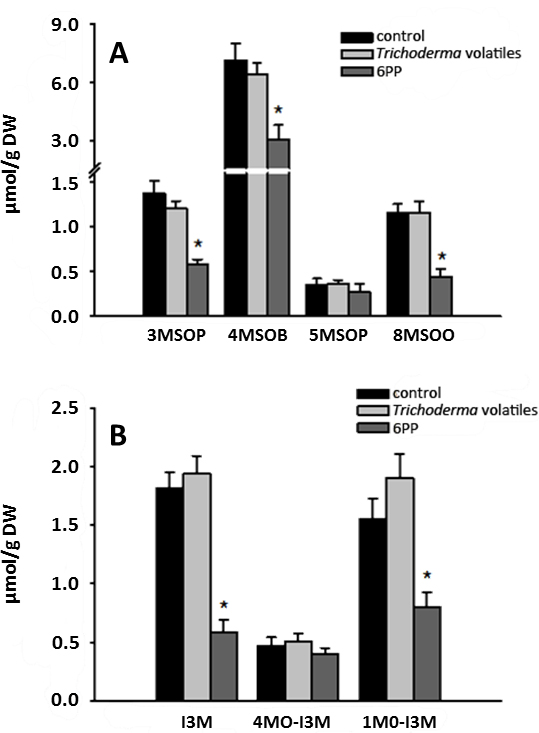

Supplement: Supplementary file 4 [file DataSheet4.ZIP › Data4/S6.JPEG]

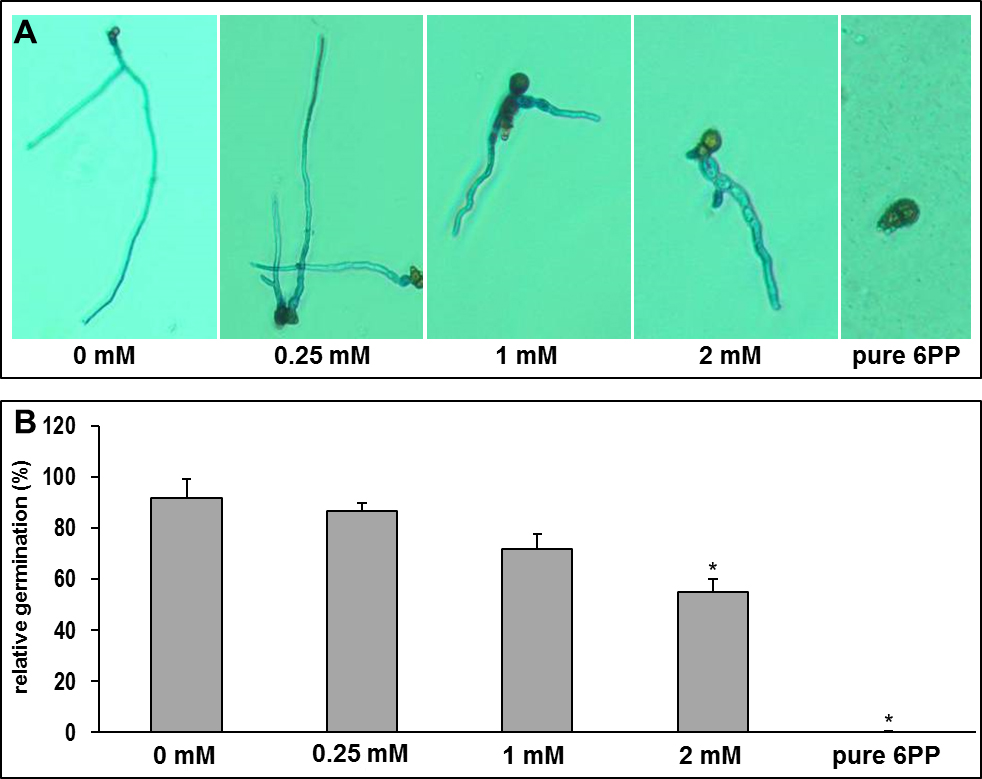

Supplement: Supplementary file 4 [file DataSheet4.ZIP › Data4/S7.JPEG]
